# Time-restricted feeding’s effect on overweight and obese patients with chronic kidney disease stages 3-4: A prospective non-randomized control pilot study

**DOI:** 10.3389/fendo.2023.1096093

**Published:** 2023-03-22

**Authors:** Bei-ni Lao, Jiang-hong Luo, Xue-yi Xu, Li-zhe Fu, Fang Tang, Wen-wei Ouyang, Xin-zhu Xu, Meng-ting Wei, Bing-jie Xiao, Lin-yi Chen, Yi-fan Wu, Xu-sheng Liu

**Affiliations:** ^1^ The Second Clinical College, Guangzhou University of Chinese Medicine, Guangzhou, China; ^2^ Department of Nephrology, The Second Affiliated Hospital of Guangzhou University of Chinese Medicine, Guangdong Provincial Hospital of Chinese Medicine, Guangzhou, China; ^3^ Chronic Disease Management Outpatient, The Second Affiliated Hospital of Guangzhou University of Chinese Medicine, Guangdong Provincial Hospital of Chinese Medicine, Guangzhou, China; ^4^ Key Unit of Methodology in Clinical Research, The Second Affiliated Hospital of Guangzhou University of Chinese Medicine, Guangdong Provincial Hospital of Chinese Medicine, Guangzhou, China; ^5^ Department of Global Public Health, Karolinska Institute, Stockholm, Sweden; ^6^ Nutritional Department, The Second Affiliated Hospital of Guangzhou University of Chinese Medicine, Guangdong Provincial Hospital of Chinese Medicine, Guangzhou, China

**Keywords:** obesity, chronic kidney disease, time-restricted feeding, TRF, renal function

## Abstract

**Background:**

Time-restricted feeding (TRF) has become a popular weight loss method in recent years. It is widely used in the nutritional treatment of normal obese people and obese people with chronic diseases such as diabetes mellitus and hypertension, and has shown many benefits. However, most TRF studies have excluded chronic kidney disease (CKD) patients, resulting in a lack of sufficient evidence-based practice for the efficacy and safety of TRF therapy for CKD. Therefore, we explore the efficacy and safety of TRF in overweight and obese patients with moderate-to-severe stage CKD through this pilot study, and observe patient compliance to assess the feasibility of the therapy.

**Methods:**

This is a prospective, non-randomized controlled short-term clinical trial. We recruited overweight and obese patients with CKD stages 3-4 from an outpatient clinic and assigned them to either a TRF group or a control diet (CD) group according to their preferences. Changes in renal function, other biochemical data, anthropometric parameters, gut microbiota, and adverse events were measured before the intervention and after 12 weeks.

**Results:**

The change in estimated glomerular filtration rate (eGFR) before and after intervention in the TRF group (Δ = 3.1 ± 5.3 ml/min/1.73m^2^) showed significant improvement compared with the CD group (Δ = -0.8 ± 4.4 ml/min/1.73m^2^). Furthermore, the TRF group had a significant decrease in uric acid (Δ = -70.8 ± 124.2 μmol/L), but an increase in total protein (Δ = 1.7 ± 2.5 g/L), while the changes were inconsistent for inflammatory factors. In addition, the TRF group showed a significant decrease in body weight (Δ = -2.8 ± 2.9 kg) compared to the CD group, and body composition indicated the same decrease in body fat mass, fat free mass and body water. Additionally, TRF shifted the gut microbiota in a positive direction.

**Conclusion:**

Preliminary studies suggest that overweight and obese patients with moderate-to-severe CKD with weight loss needs, and who were under strict medical supervision by healthcare professionals, performed TRF with good compliance. They did so without apparent adverse events, and showed efficacy in protecting renal function. These results may be due to changes in body composition and alterations in gut microbiota.

## Introduction

Nutritional therapy for chronic kidney disease (CKD) has received increasing attention in recent years. Previous studies may have focused on protein energy expenditure in patients with CKD, but with lifestyle changes, the prevalence of obesity has gradually increased (1). At present, there is a growing population of obese CKD patients. Obesity is a chronic metabolic disease caused by multiple factors. It is an independent risk factor for the development and progression of CKD (2). Commonly reported weight loss strategies for obese patients with CKD include lifestyle changes such as dietary habits, exercise, drug therapy, and bariatric surgery (3). Nutritional therapy offers a more effective conservative treatment for obese CKD patients in the moderate and severe stages (4).

Intermittent fasting has been an attention-grabbing approach to successful weight reduction for obese adults in recent years. Previous studies have shown that intermittent fasting methods (5–7), time-restricted feeding (8, 9), and caloric restriction (10) reduce body weight in obese patients. The mechanism may be associated with metabolic benefits (11), but more likely due to caloric restriction (12). Nephropathy studies have achieved the same effect by adopting a very low-calorie diet as a weight-loss method for hemodialysis patients (13). Conversely, in non-dialysis patients, several studies have adopted caloric restriction combined with exercise intervention (14–16), demonstrating the benefits of weight loss and improved glucose and lipid metabolism, but no significant benefit on renal function. Moreover, most study populations have consisted of primarily patients in the early stages of CKD, and there has been a lack of relevant studies of patients with severe CKD.

Time-restricted feeding is one form of intermittent fasting popular for its benefits in weight management, improving cardiovascular indicators, and promoting insulin resistance (17). Nevertheless, probably because of the principle of requiring an adequate caloric intake, low-protein diets for CKD patients conflict with the demands of fasting. Thus, most TRF studies have listed CKD patients as one of the exclusion criteria, resulting in a deficiency of medical evidence for the efficacy and safety of current TRF therapy for CKD. Therefore, evidence from clinical studies is needed to determine whether CKD patients can tolerate this therapy, and how they comply. For these purposes, we proposed investigating the efficacy and safety of TRF in overweight and obese moderate-to-severe CKD patients, and evaluated its clinical feasibility through this pilot study.

Meanwhile, chronic low-grade inflammation in adipose tissue had been shown to be an important contributor to metabolic disorders, and thus obesity is also considered an inflammatory immune disease (18). Animal studies have shown that dietary modifications such as fasting can improve inflammatory diseases (19). Similarly, TRF has been shown to improve inflammation in animal studies, helping to combat inflammatory and metabolic diseases (20). Clinical evidence suggests that fasting and caloric restriction can reduce systemic inflammation and clinical symptoms (21, 22). TRF has also been shown to reduce inflammatory symptoms in patients with polycystic ovary syndrome (23). However, there are few studies on TRF for other chronic diseases combined with abnormal metabolic disorders. On the other hand, metabolic diseases such as obesity or dyslipidemia in the moderate-to-severe stages of CKD are commonly associated with a microinflammation state. Therefore, in our study, we evaluated whether TRF ameliorates inflammation in patients with moderate-to-severe CKD combined with overweight or obese by detecting inflammatory indicators.

The relationship between CKD and gut microbiota has always been a research hotspot. Based on the gut-kidney axis theory (24), CKD interacts with gut microbiota. CKD metabolic wastes affect the composition of gut microbiota, in turn, microbial metabolism produces uremic toxins that affect the host. Protein fermentation by gut microbiota produces numerous toxic metabolites, including p-cresyl sulfate and indoxyl sulfate. The destruction of gut barrier function in CKD patients leads to the transfer of endotoxins and harmful bacterial metabolites to systemic circulation. This causes a strong immune inflammatory response in the body, leading to uremia, promoting the progression of CKD and inducing cardiovascular diseases and other related comorbidities. Changes in gut microbiota in obese patients lead to energy metabolism imbalance, fat deposition and insulin resistance (25). The bioactive metabolites short-chain fatty acids and conjugated fatty acids produced by the gut microbiota are diet-dependent (26), which in turn can regulate the body’s appetite. Diet structure and dietary patterns in nutritional therapy can cause gut microbiota changes, improve flora imbalance, and thus affect host metabolism (27). Animal studies have shown that TRF exerts metabolic benefits by restoring the circadian rhythm of gut microbiota (28, 29). However, data on the effect of TRF on the gut microbiology of patients are not only scarce, but also the conclusions have different directions (30–32), making it difficult to generalize. Therefore, changes in gut microbiota were included in the observations in this study.

## Materials and methods

### Patient population

This is a prospective, non-randomized, controlled exploratory intervention study conducted in the nephrology outpatient center at Guangdong Provincial Hospital of Chinese Medicine in Guangzhou, China. We recruited patients through an extensive advocacy recruitment approach, such as advertisements posted on various WeChat official accounts (a popular Chinese social media app), recruitment posters in our research unit’s building and phone calls. However, due to the novelty of the dietary strategy itself, fewer patients with CKD had heard of it and were therefore reluctant to be the first to try it. Additionally, because of the epidemic’s effect, fewer patients were willing to participate. The protocol was conducted in accordance with the Declaration of Helsinki and approved by the Ethics Committee at the Guangdong Provincial Hospital of Traditional Chinese Medicine (approval number: YF2021-142; approval date: July 12, 2021). All patients were enrolled in August 2021, and the trial ended in March 2022. The study was registered with ClinicalTrails.gov, NCT05037747.

Inclusion criteria: a) 18-65 years old; b) CKD stages 3-4 [estimated glomerular filtration rate (eGFR) 15-59 mL/min/1.73m^2^], eGFR was calculated using the Chronic Kidney Disease Epidemiology Collaboration (CKD-EPI) formula; c) overweight or obese with body mass index (BMI) ≥ 25 kg/m^2^, according to the classifications adopted by the World Health Organization (WHO) (33); d) good reading and comprehension skills, the ability to operate a smartphone, and no communication barriers.

Exclusion criteria: a) pregnant or breastfeeding patients; b) acute or active diseases such as acute infection or active bleeding; c) end-stage diseases, complex diseases with severe decompensation such as decompensated liver cirrhosis, malignant tumors, severe cardiopulmonary disease, or severe hematopoietic diseases; d) severe hypertension (systolic blood pressure ≥ 200 mmHg, diastolic blood pressure ≥ 120 mmHg) and uncontrollable blood pressure; e) within three months after major surgery such as laparotomy; f) mentally ill patients; g) those who are taking hormones or immunosuppressants; h) those who are participating in other investigations, or are undergoing other diet therapy or weight loss therapy; i) vegetarians; j) patients with type 1 or type 2 diabetes taking insulin. The study flow diagram is shown in **Supplementary Figure 1**.

### Study design

88 patients were evaluated as eligible, 26 were excluded because they did not meet the inclusion criteria, 28 were excluded because they did not want to participate, and 6 were excluded for other reasons. Ultimately, only 28 were included in the study, and were assigned to either the TRF group or the CD group according to their preferences. The CD group received a high-quality low-protein diet with no restrictions on what time they could eat each day, following their daily routines. In contrast, on the basis of a high-quality low-protein diet, the TRF group adopted time-restricted feeding. Patients were required to consume three meals within 8 hours. The start time of these 8 hours was stipulated between 7:00 a.m. and noon. No calorie-containing food could be consumed during the fasting period, and only drinking water and non-caloric beverages were allowed. Meanwhile, both groups maintained conventional treatment according to the guidelines. Participants and researchers were informed of the group assignments.

The research group consisted of specialized nephrologists, nurse practitioners, and nutritionists. Both groups of participants received a detailed dietary guide and daily recommended recipes at enrollment, which the researchers explained in detail. During the study period, researchers contacted participants on-line every two weeks to inquire about their diets and to offer appropriate instructions to ensure that participants followed the established dietary intake regimen as close as possible. Participants were trained to record daily diaries. In Week 6 we scheduled a face-to-face follow-up visit. We assessed patients for adverse events at Week 6 and Week 12. At the same time, we used visual analogue scale (VAS) scores (ranging from 0-10, with 0 indicating none, and a higher score indicating worse symptoms) to evaluate the discomfort symptoms, including hunger, fatigue, satiety, flatulence, nausea, vomiting, constipation, diarrhea, bad breath, dry mouth, dizziness, headache, weakness, irritability, unhappiness, depression, and anxiety.

The primary outcome indicator was the change in eGFR. Secondary outcomes included changes in biochemistry, anthropometric parameters and gut microbiota.

### Biochemistry

At baseline and at the 12-week time-point return visit, after a fast of at least 8 hours, blood samples were collected from arm veins between 8:00 a.m. and 9:00 a.m. and transferred to the unit’s laboratory. Renal function indicators, nutritional and lipid indicators, fasting blood glucose, inflammation indicators, β-hydroxybutyric acid, blood routine, liver function, and electrolytes were tested according to the criteria.

### Anthropometric parameters

Arm circumference, triceps skinfold thickness and waist circumference were measured at baseline and 12 weeks. To ensure consistency, measurements were taken by the same trained researcher with a soft ruler. Triceps skinfold thickness was measured with a triceps skinfold caliper with a precise measurement of up to 80.0 mm. For arm circumference measurement, subjects stood naturally with their arms bare, and uniformly measured the right upper arm. From the acromion to the olecranon’s midpoint was accurate to 0.1 cm. Triceps skinfold thickness was measured at 2 cm below the midpoint, as identified by the above method, pinching the thickness of the skin and subcutaneous tissue, and measuring with a caliper to an accuracy of 0.1cm. Waist circumference was measured at the midpoint of the anterior superior iliac spine and the lower margin of the 12th rib, accurate to 0.1 cm, with subjects standing with the waist and abdomen exposed. Handgrip strength was measured with a grip dynamometer averaging three readings expressed in kilograms. Heart rate, systolic blood pressure and diastolic blood pressure were measured at baseline and after the intervention.

Bioelectrical impedance analysis (BIA) was utilized to assess body composition (InBody 770; Biospace Co., Ltd, Seoul, Korea). Both groups’ body compositions were measured while patients were wearing light singlets, no shoes or socks, with empty bladders, and calibrated instruments. Measurement items included: weight, body mass index, waist-to-hip ratio, fat free mass, body fat mass, percentage of body fat, visceral fat area, fat mass index, soft lean mass, skeletal muscle mass, total body water, intracellular water and extracellular water.

### Adherence

Patients in both groups submitted a three-day diet record and a daily eating time record at the 12-week return visit. Researchers determined patient compliance in the TRF group based on the requirement of an 8-hour restricted feeding time period. In terms of patient dietary records, the analysis was performed in accordance with China Food Composition (2nd Edition) (34). Whether patients had been on a low-protein diet was measured by dietary protein intake (DPI) corrected for standard weight. We used the standard weight calculation method for Asian bodies: (male) standard weight = [height (cm) - 100] × 0.9 (kg); (female) standard weight = [height (cm) - 100] × 0.9 (kg) - 2.5 (kg). Protein intake was in accordance with the CKD nutritional standards, in which the recommended protein intake for non-dialysis patients with CKD stages 3-5 was 0.6-0.8g/kg/d^1^ (35).

### Exercise volume assessment

To avoid bias in the results caused by differences in the amount of exercise, we registered the patients’ step counts for measurement, and asked them to bring the measurement records for statistics at the 12-week return visit. Exercise was recorded on two days of the week (from Monday to Friday) and one day on the weekend over the period of a month. It was based the number of walking steps on these three days.

### Fecal collection

Patients retained fresh feces at baseline and at the 12-week return visit. Approximately 5 g of feces was collected with a sterilized scoop and placed in the intestinal microbe collection tube. The feces was immediately stored in an -80℃ freezer for later unified analysis.

### Microbiota analysis

DNA was obtained from fecal samples using a MagPure Stool DNA KF kit B (Magen, China) following the manufacturer’s instructions. It was quantified with a Qubit Fluorometer by using a Qubit^®^ dsDNA BR Assay kit (Invitrogen, USA) and was checked on 1% agarose gel. To construct the 16S rDNA library, the hypervariable region V4 of the 16S rRNA gene was amplified with PCR primers 515F (5’- GTGCCAGCMGCCGCGGTAA -3’) and 806R (5’- GGACTACHVGGGTWTCTAAT-3’). Both forward and reverse primers were tagged with Illumina adapter, pad, and linker sequences. PCR enrichment was performed in a 50 μL reaction containing a 30ng template, fusion PCR primer, and PCR master mix. The PCR products were purified with AmpureXP beads and eluted in the Elution buffer. Libraries were qualified by the Agilent 2100 bioanalyzer (Agilent, USA). The validated libraries were used for sequencing on the Illumina HiSeq2500 platform (BGI, Shenzhen, China) following the standard pipelines of Illumina and generating 2 × 300 bp paired-end reads. The sequencing results were filtered to remove any adaptors or low-quality or ambiguous bases, and then paired-end reads were added to tags by the Fast Length Adjustment of SHort reads program (FLASH, v.1.2.11) (36) to get the tags. Operational taxonomic units (OTUs) with a 97% similarity cutoff were clustered using UPARSE version 7.0.1090 (37). OTU representative sequences were taxonomically classified using Ribosomal Database Project (RDP) Classifier v.2.2 with a minimum confidence threshold of 0.6.

### Statistical analysis

This study was conducted as an exploratory pilot study, with CKD patients recruited for the study whenever possible. Therefore, there was no formal calculation of sample size. Instead, the available data were used to determine the sample size. Statistical data were analyzed using SPSS (v.26; SPSS, Inc), with qualitative variables expressed as rates or percentages and continuous variables described by means ± standard deviation (SD) or median and 25th-75th interquartiles. We calculated the variables’ changes (deltas) as final value minus baseline value. For between-group comparisons, we used an independent Student’s t-test for data with a normal distribution, and a Mann-Whitney U-test for data with a non-normal distribution. For within-group comparisons, we used a paired t-test for normally distributed data, and a Wilcoxon signed-rank test for nonnormally distributed data. Qualitative data proportion or categorical variables were measured by a chi-square test or Fisher’s exact test. Due to the small number of missing value, the missing data was imputed using mean imputation. We adjusted for confounding factors using multiple linear regression, and selected the ENTER method for screening variables. Any variables with uneven baselines, clinically relevant variables with *p* < 0.05 for one-way analysis, or clinical variables with effects on eGFR were included in the multiple linear regression model. The variables included were selected to ensure a streamlined final model, accounting for the small number of available events. A two-sided test was used for the statistical test, and *p* < 0.05 was regarded as statistically significant.

The gut microbiota alpha diversity index (chao1 index) used a t-test. Statistical significance of the beta diversity weighted and un-weighted UniFrac matrices were measured by ANOSIM. Alpha and beta diversity, as well as Principal Coordinate Analysis (PCoA), were analyzed using QIIME (v.1.8.0) (38). Linear discriminant analysis (LDA) analysis was conducted by LEfSe.

## Results

### Trial participants

28 patients were assigned to either the TRF group (n=13) or the CD group (n=15), and one patient in the CD group dropped out. The patients were 51.9% male, mean age 52.2 ± 9.6 years, mean weight 76.2 ± 10.8 kg, mean baseline eGFR 40.6 ± 10.8 mL/min/1.73m^2^. The two groups of patients were similar at baseline except for diastolic blood pressure and fasting blood glucose. A slightly lower eGFR (*p* = 0.074) was observed in the TRF group at baseline, but it was not statistically significant. The baseline characteristics of the patients are shown in **Table 1**.

**Table 1:** Characteristics of the patients at the beginning of the intervention.

Variable	TRF group (n = 13)	CD group (n = 14)	*p*-value
Sex (male)%	7 (53.9)	7 (50.0)	1.000
Age (years)	51.8 ± 7.7	52.5 ± 11.3	0.847
Primary renal disease			
Diabetes mellitus	4 (30.8)	5 (35.7)	1.000
Hypertension	1 (7.7)	2 (14.3)	1.000
Glomerulonephritis	2 (15.4)	3 (21.4)	1.000
Other/unknown	6 (46.2)	4 (28.6)	0.585
CKD stage			
Stage 3	9 (69.2)	12 (85.7)	0.385
Stage 4	4 (30.8)	2 (14.3)	
Comorbidities			
Diabetes mellitus	4 (30.8)	7 (50.0)	0.440
Hypertension	12 (92.3)	10 (71.4)	0.326
Hyperlipidemia	8 (61.5)	8 (57.1)	1.000
Hyperuricemia	10 (76.9)	10 (71.4)	1.000
Cardiovascular diseases	2 (15.4)	3 (21.4)	1.000
SBP (mmHg)	130.7 ± 11.3	125.4 ± 15.7	0.325
DBP (mmHg)	80.9 ± 6.0	72.2 ± 9.1	**0.007**
HR (beats/min)	71.5 ± 10.7	76.1 ± 10.9	0.279
Weight (kg)	79.4 ± 10.6	73.3 ± 10.5	0.142
BMI (kg/m^2^)	29.3 ± 2.3	28.0 ± 2.4	0.151
eGFR (ml/min/1.73m^2^)	36.7 ± 10.5	44.2 ± 10.2	0.074
SCR (μmol/L)	173.9 ± 52.5	144.1 ± 34.5	0.092
BUN (mmol/L)	9.0 ± 3.2	8.9 ± 2.6	0.914
UA (μmol/L)	450 ± 110.7	399.1 ± 100.6	0.089
PCR (g/g)	0.724 ± 0.662	0.601 ± 0.466	0.580
Cys-C (mg/L)	2.4 ± 0.8	2.2 ± 0.4	0.981

Data are shown as mean ± standard deviation (SD) or number and proportion. TRF, time-restricted feeding; CD, control diet; CKD, chronic kidney disease; SBP, systolic blood pressure; DBP, diastolic blood pressure; HR, heart rate; BMI, body mass index; eGFR, estimated glomerular filtration rate; SCR, serum creatinine; BUN, blood urea nitrogen; UA, uric acid; PCR, urinary protein/creatinine; Cys-C, cystatin-C. *p* values < 0.05 are in boldface.

### Kidney function improvement

**Figure 1:**
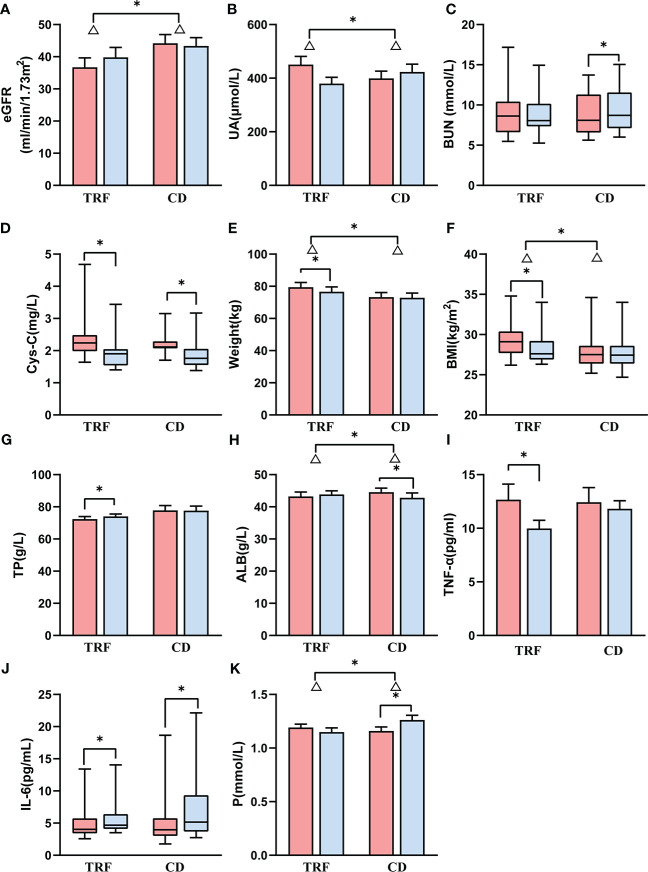
Comparison of renal function and other biochemical parameters between groups. Data following a normal distribution are presented as error bar plots; data not following a normal dis-tribution are presented as box-and-whisker plots. Red represents before intervention, and blue represents after intervention. TRF: time-restricted feeding; CD: control diet; **(A)** estimated glomerular filtration rate; **(B)** uric acid; **(C)** blood urea nitrogen; **(D)** cystatin-C; **(E)** weight; **(F)** body mass index; **(G)** total protein; **(H)** albumin; **(I)** tumor necrosis factor alpha; **(J)** interleukin 6; **(K)** serum phosphorus. Δ represents the difference, * represents *p* < 0.05.

Of note, the TRF group (Δ = 3.1 ± 5.3 ml/min/1.73m^2^) had a greater increase in eGFR than the CD group (Δ = -0.8 ± 4.5 ml/min/1.73m^2^) (*p* = 0.049, **Figure 1A**) for the primary outcome indicator. In addition, among the remaining secondary outcome indicators, the changes in uric acid were more variable; the decrease in the TRF group (Δ = -70.8 ± 124.2 μmol/L) was greater than that of the CD group (Δ = 24.3 ± 76.5 μmol/L) (*p* = 0.023, **Figure 1B**). Additionally, blood urea nitrogen was significantly higher (*p* = 0.030, **Figure 1C**) in the CD group (Δ = 0.5 ± 0.7 mmol/L) than in previous results. In parallel with eGFR, serum creatinine in the TRF group (Δ = -10.9 ± 21.9 μmol/L) decreased slightly more than the CD group (Δ = -1.3 ± 14.9 μmol/L) (*p* = 0.101). In addition, although cystatin-C between the TRF group (Δ = -0.5 ± 0.4 mg/L, *p* = 0.003) and the CD group (Δ = -0.3 ± 0.3 mg/L, *p* = 0.003) were significantly lower than after the intervention, there was no difference between the groups (*p* = 0.381, **Figure 1D**).

### Weight loss

The TRF group (Δ = -2.8 ± 2.9 kg) showed a significant decrease in body weight (*p* = 0.004), which was greater than that of the CD group (Δ = -0.4 ± 1.4 kg) (*p*=0.010, **Figure 1E**). Similarly, the BMI of the TRF group (Δ = -1.1 ± 1.1 kg/m^2^) also decreased significantly (*p* = 0.005), and the reduction was more significant than that of the CD group (Δ = -0.2 ± 0.6 kg/m^2^) (*p* = 0.013, **Figure 1F**).

### Nutritional improvement

The total protein in the TRF group (Δ = 1.7 ± 2 g/L) significantly increased (*p* = 0.039), and slightly decreased in the CD group (Δ = -0.1 ± 3.6 g/L), with no significant difference between the groups (*p* = 0.136, **Figure 1G**). On the other hand, the albumin of the CD group (Δ = -1.7 ± 3.0 g/L) decreased significantly (*p* = 0.003) and was significantly lower compared to the TRF group (Δ = 0.6 ± 2.5 g/L) (*p* = 0.034, **Figure 1H**).

### Other biochemical results

The effect of TRF on inflammatory factors was inconclusive. Tumor necrosis factor alpha (TNF-α) in the TRF group (Δ = -2.7 ± 3.5 pg/ml, *p* = 0.017, **Figure 1I**) decreased significantly from the baseline, but did not differ from the CD group (Δ = -0.6 ± 3.8 pg/ml) (*p* = 0.156), whereas interleukin 6 (IL-6) increased in the TRF group [Δ = 0.7 (0.4, 1.4) pg/mL, *p* = 0.028] and the CD group [Δ = 1.2 (-0.2, 3.4) pg/mL, *p* = 0.019] compared with the pre-intervention (**Figure 1J**), and there was no effect on C-reactive protein (CRP).

Moreover, there was a significant increase in serum phosphorus in the CD group (Δ = 0.11 ± 0.11 mmol/L, *p* = 0.004). This showed that there was a significant difference compared with the TRF group (Δ = -0.04 ± 0.18 mmol/L) (*p* = 0.015, **Figure 1K**).

There were no significant differences in the changes in serum calcium, serum sodium, serum potassium or serum chloride. This suggested that TRF had not impaired the hydroelectrolytic balance. Regrettably, we found no amelioration in blood pressure, fasting blood glucose or blood lipids with TRF. There was also no influence with regard to heart rate, β- hydroxybutyric acid, liver function, or blood routine between the TRF group and the CD group.

### Body composition and anthropometrics

**Figure 2:**
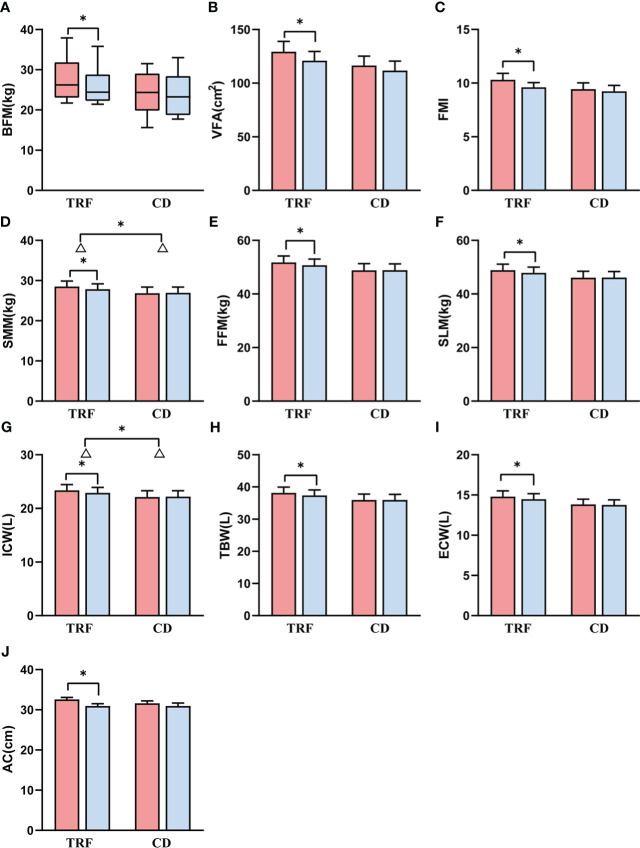
Comparison of body composition and anthropometrics between groups. Data following a normal distribution are presented as error bar plots, and data not following a normal distribution are presented as box-and-whisker plots. Red represents before intervention, and blue represents after intervention. TRF: time-restricted feeding; CD: control diet; **(A)** body fat mass; **(B)** visceral fat area; **(C)** fat mass index; **(D)** skeletal muscle mass; **(E)** fat free mass; **(F)** soft lean mass; **(G)** intracellular water; **(H)** total body water; **(I)** extracellular water; **(J)** arm circumference. Δ is the difference; * represents *p* < 0.05.

In terms of body fat, the body fat mass, visceral fat area, and fat mass index in the TRF group (Δ = -1.8 ± 2.4 kg, *p* = 0.014, Δ = -8.4 ± 12.1 cm^2^, *p* = 0.028, Δ = -0.7 ± 0.9, *p* = 0.018, respectively) decreased significantly, but were not substantially different from those in the CD group (Δ = -0.4 ± 1.8 kg, Δ = -4.8 ± 16.5 cm^2^, Δ = -0.2 ± 0.8, respectively) (*p* = 0.119, **Figure 2A**, *p* = 0.523, **Figure 2B**, *p* = 0.146, **Figure 2C**).

However, for muscle, skeletal muscle mass in the TRF group (Δ = -0.7 ± 0.8 kg, *p* = 0.010) decreased significantly more than that in the CD group (Δ = 0.1 ± 1.0 kg) (*p* = 0.042, **Figure 2D**). Fat free mass and soft lean mass in the TRF group (Δ = -1.1 ± 1.4 kg, *p* = 0.016, Δ = -1.0 ± 1.2 kg, *p* = 0.012) also showed a significant decrease, which was not significantly different from that in the CD group (Δ = 0.0 ± 1.6 kg, Δ = 0.0 ± 1.5 kg) (*p* = 0.066, **Figure 2E**, *p* = 0.055, **Figure 2F**).

In regard to body water, intracellular water in the TRF group (Δ = -0.5 ± 0.6 L, *p* = 0.011) decreased significantly, and the decline had a greater degree of statistical significance than that in the CD group (Δ = 0.1 ± 0.8 L) (*p* = 0.050, **Figure 2G**). The total body water and extracellular water in the TRF group (Δ = -0.8 ± 1.0 L, *p* = 0.010, Δ = -0.3 ± 0.4 L, *p* = 0.020) also decreased significantly, but was not statistically different from that of the CD group (Δ = 0.1 ± 1.1 L, Δ = -0.1 ± 0.4 L) (*p* = 0.054, **Figure 2H**, *p* = 0.142, **Figure 2I**).

Anthropometrically, there was a statistical decrease in upper arm circumference in the TRF group (Δ = -1.6 ± 1.2 cm, *p* < 0.001), but there was no significant difference with the CD group (Δ = -0.6 ± 1.4 cm) (*p* = 0.068, **Figure 2J**). Despite not reaching significance, the percentage of body fat (Δ = -1.0 ± 2.0, *p* = 0.055) and waist circumference (Δ = -1.9 ± 3.2, *p* = 0.051) showed a tendency to decrease in the TRF group. Nevertheless, there were no significant differences in triceps skin fold thickness, waist-to-hip ratio, hand grip strength, or conicity index between the TRF group and the CD group. Differences between two groups before and after intervention are shown in **Tables 2**, **3**. Important biochemical indicators and reference values are shown in **Supplementary Table 1**.

**Table 2:** Differences between the TRF group and the CD group, before and after treatment.

Variable	TRF (n = 13)			CD (n = 14)		
	Pre	Post	*p*-value	Pre	Post	*p*-value
eGFR (ml/min/1.73m^2^)	36.7 ± 10.5	39.8 ± 11.1	0.056	44.2 ± 10.2	43.4 ± 9.7	0.513
SCR (μmol/L)	173.9 ± 52.5	163.0 ± 52.1	0.097	144.1 ± 34.5	145.4 ± 32.3	0.752
BUN (mmol/L)	9.0 ± 3.2	9.1 ± 2.9	0.889	8.9 ± 2.6	9.3 ± 2.8	**0.030**
UA (μmol/L)	450 ± 110.7	379.9 ± 84.6	0.062	399.1 ± 100.6	423.4 ± 108.5	0.256
PCR (g/g)	0.724 ± 0.662	0.795 ± 0.720	0.875	0.601 ± 0.466	0.730 ± 0.662	0.221
Cys-C (mg/L)	2.4 ± 0.8	1.9 ± 0.5	**0.003**	2.2 ± 0.4	1.9 ± 0.5	**0.003**
Weight (kg)	79.4 ± 10.6	76.6 ± 10.8	**0.004**	73.3 ± 10.5	72.9 ± 10.8	0.314
BMI (kg/m^2^)	29.3 ± 2.3	28.2 ± 2.1	**0.007**	28.0 ± 2.4	27.8 ± 2.3	0.231
ALB (g/L)	43.2 ± 5.0	43.9 ± 4.1	0.485	44.6 ± 4.7	42.8 ± 5.6	**0.003**
TP (g/L)	72.4 ± 5.8	74.1 ± 5.4	**0.039**	77.8 ± 11.3	77.7 ± 10.6	0.975
IL-6 (pg/mL)	5.1 ± 2.9	5.7 ± 2.9	**0.028**	5.2 ± 4.2	7.0 ± 5.1	**0.019**
TNF-α (pg/ml)	12.7 ± 5.3	10.0 ± 2.8	**0.017**	12.4 ± 5.1	11.8 ± 2.8	0.550
CRP (mg/L)	4.5 ± 4.7	2.8 ± 2.7	0.182	7.8 ± 17.6	9.1 ± 16.5	0.279
Na (mmol/L)	140.4 ± 1.8	140.7 ± 1.9	0.501	139.8 ± 2.2	139.4 ± 2.6	0.469
P (mmol/L)	1.19 ± 0.11	1.15 ± 0.14	0.695	1.16 ± 0.14	1.26 ± 0.16	**0.004**
Ca (mmol/L)	2.35 ± 0.08	2.40 ± 0.10	0.129	2.39 ± 0.13	2.39 ± 0.10	0.905
K (mmol/L)	4.24 ± 0.56	4.43 ± 0.44	0.196	4.45 ± 0.42	4.47 ± 0.48	0.875
Cl (mmol/L)	105.6 ± 2.6	105.0 ± 2.1	0.173	104.8 ± 2.6	104.8 ± 3.1	0.529
SBP (mmHg)	130.7 ± 11.3	130.9 ± 14.7	0.946	125.4 ± 15.7	127.9 ± 12.6	0.228
DBP (mmHg)	80.9 ± 6.0	79.7 ± 10.7	0.520	72.2 ± 9.1	71.6 ± 11.5	0.747
HR (beats/min)	71.5 ± 10.7	75.3 ± 11.2	0.404	76.1 ± 10.9	77.8 ± 9.3	0.586
FBG (mmol/L)	4.8 ± 0.7	5.2 ± 1.0	0.263	6.6 ± 2.9	5.7 ± 1.0	0.116
TC (mmol/L)	4.72 ± 1.43	4.30 ± 0.85	0.285	4.19 ± 0.91	4.33 ± 1.25	0.354
TG (mmol/L)	1.7 ± 0.8	1.6 ± 0.7	0.279	2.0 ± 1.0	2.0 ± 1.7	0.463
LDL (mmol/L)	3.05 ± 1.19	2.60 ± 0.77	0.220	2.43 ± 0.68	2.52 ± 0.88	0.485
HDL (mmol/L)	1.14 ± 0.28	1.23 ± 0.30	0.115	1.11 ± 0.30	1.15 ± 0.30	0.278
β-HB (mmol/L)	0.07 ± 0.03	0.08 ± 0.05	0.637	0.09 ± 0.09	0.11 ± 0.13	0.407
ALT (U/L)	24.2 ± 23.7	23.8 ± 24.0	0.529	22.6 ± 17.9	17.6 ± 11.1	**0.013**
AST (U/L)	19.1 ± 8.2	19.8 ± 8.6	0.454	24.4 ± 12.1	21.4 ± 7.2	0.340
WBC (10^9/L)	6.0 ± 1.8	5.9 ± 1.7	0.718	7.4 ± 1.7	7.3 ± 2.1	0.788
NEUT%	60.6 ± 7.8	61.7 ± 6.5	0.574	64.0 ± 8.8	62.5 ± 6.3	0.438
LYM%	29.0 ± 7.5	28.0 ± 5.8	0.507	25.5 ± 7.7	25.8 ± 5.6	0.854
RBC (10^12/L)	4.5 ± 0.6	4.6 ± 0.5	0.564	4.4 ± 0.7	4.4 ± 0.8	0.681
HB (g/L)	129.2 ± 20.8	131.9 ± 18.8	0.294	130.9 ± 22.5	130.3 ± 25.1	0.278
PLT (10^9/L)	228.4 ± 31.4	231.6 ± 38.3	0.345	261.7 ± 72.0	273.0 ± 77.6	0.109
BFM (kg)	27.7 ± 5.7	25.9 ± 4.8	**0.014**	24.5 ± 5.2	24.0 ± 5.3	0.470
VFA (cm^2^)	129.3 ± 34.9	120.9 ± 31.3	**0.028**	116.4 ± 32.8	111.7 ± 33.2	0.300
FMI	10.3 ± 2.2	9.6 ± 1.6	**0.018**	9.4 ± 2.2	9.2 ± 2.1	0.340
PBF%	34.9 ± 5.6	33.9 ± 4.3	0.055	33.6 ± 6.8	33.1 ± 6.0	0.593
FFM (kg)	51.8 ± 8.6	50.7 ± 8.3	**0.016**	48.8 ± 9.3	48.9 ± 8.9	0.921
SLM (kg)	48.9 ± 8.1	47.9 ± 7.9	**0.012**	46.1 ± 8.9	46.1 ± 8.5	0.928
SMM (kg)	28.5 ± 5.0	27.8 ± 4.8	**0.010**	26.8 ± 5.7	26.9 ± 5.4	0.740
TBW (L)	38.1 ± 6.4	37.3 ± 6.2	**0.010**	35.9 ± 6.9	35.9 ± 6.5	1.000
ICW (L)	23.4 ± 3.8	22.9 ± 3.7	**0.011**	22.1 ± 4.4	22.2 ± 4.2	0.745
ECW (L)	14.8 ± 2.6	14.5 ± 2.5	**0.020**	13.8 ± 2.5	13.8 ± 2.4	0.545
AC (cm)	32.6 ± 1.9	31.0 ± 2.0	**0.000**	31.6 ± 2.3	31.0 ± 2.7	0.111
TSF (cm)	2.2 ± 0.4	2.1 ± 0.6	0.714	2.2 ± 0.5	2.2 ± 0.8	0.972
WC (cm)	100.8 ± 8.3	98.8 ± 7.6	0.051	95.1 ± 9.7	95.2 ± 8.6	0.932
WHR	0.91 ± 0.08	0.91 ± 0.07	1.000	0.90 ± 0.05	0.90 ± 0.06	0.833
Left HGS (kg)	28.9 ± 8.4	27.7 ± 9.1	0.266	29.0 ± 9.1	28.0 ± 9.8	0.070
Right HGS (kg)	31.9 ± 11.1	29.9 ± 10.5	0.102	31.6 ± 10.9	30.4 ± 10.7	0.195
CI	1.33 ± 0.07	1.33 ± 0.06	0.897	1.30 ± 0.09	1.30 ± 0.06	0.598

Data are shown as mean ± standard deviation (SD). TRF, time-restricted feeding; CD, control diet; eGFR, estimated glomerular filtration rate; SCR, serum creatinine; BUN, blood urea nitrogen; UA, uric acid; PCR, urinary protein/creatinine; Cys-C, cystatin-C. BMI, body mass index; ALB, albumin; TP, total protein; IL-6, interleukin 6; TNF-α, tumor necrosis factor alpha; CRP, C-reactive protein; Na, serum sodium; P, serum phosphorus; Ca, serum calcium; K, serum potassium; Cl, serum chlorine; SBP, systolic blood pressure; DBP, diastolic blood pressure; HR, heart rate; FBG, fasting blood glucose; TC, total cholesterol; TG, triglycerides; LDL, low-density lipoprotein; HDL, high-density lipoprotein; β-HB, beta hydroxybutyric acid; ALT, alanine transaminase; AST, aspartate transaminase; WBC, white blood cells; NEUT%, neutrophilic granulocyte percentage; LYM%, lymphocyte percentage; RBC, red blood cells; HB, hemoglobin; PLT, platelets; BFM, body fat mass; VFA, visceral fat area; FMI, fat mass index; PBF, percentage of body fat; FFM, fat free mass; SLM, soft lean mass; SMM, skeletal muscle mass; TBW, total body water; ICW, intracellular water; ECW, extracellular water; AC, arm circumference; TSF, triceps skinfold thickness; WC, waist circumference; WHR, waist-to-hip ratio; HGS, handgrip strength; CI, conicity index. *p* values < 0.05 are in boldface.

**Table 3:** Comparison of changes between the TRF group and the CD group.

Variable	ΔTRF (n=13)	ΔCD (n=14)	*p*-value
eGFR (ml/min/1.73m^2^)	3.1 ± 5.3	-0.8 ± 4.5	**0.049^a^**
SCR (μmol/L)	-10.9 ± 21.9	1.3 ± 14.9	0.101^a^
BUN (mmol/L)	0.1 ± 2.3	0.5 ± 0.7	0.559^a^
UA (μmol/L)	-70.8 ± 124.2	24.3 ± 76.5	**0.023^a^**
PCR (g/g)	0.007 (-0.089, 0.328)	0.052 (-0.037, 0.204)	0.497^b^
Cys-C (mg/L)	-0.5 ± 0.4	-0.3 ± 0.3	0.381^a^
Weight (kg)	-2.8 ± 2.9	-0.4 ± 1.4	**0.010^a^**
BMI (kg/m^2^)	-1.1 ± 1.1	-0.2 ± 0.6	**0.013^a^**
ALB (g/L)	0.6 ± 2.5	-1.7 ± 3.0	**0.034^a^**
TP (g/L)	1.7 ± 2.5	-0.1 ± 3.6	0.136^a^
IL-6 (pg/mL)	0.7 (0.4, 1.4)	1.2 (-0.2, 3.4)	0.467^b^
TNF-α (pg/ml)	-2.7 ± 3.5	-0.6 ± 3.8	0.156^a^
CRP (mg/L)	-0.5 (-3.4, 0.9)	0.5 (-0.5, 1.2)	0.139^b^
Na (mmol/L)	0.3 ± 1.8	-0.4 ± 2.3	0.367^a^
P (mmol/L)	-0.04 ± 0.18	0.10 ± 0.11	**0.015^a^**
Ca (mmol/L)	0.05 ± 0.11	-0.00 ± 0.11	0.218^a^
K (mmol/L)	0.20 ± 0.42	0.02 ± 0.37	0.261^a^
Cl (mmol/L)	-0.5 (-2.0, 0.2)	0.2 (-0.6, 1.2)	0.120^b^
SBP (mmHg)	0.2 ± 12.0	2.6 ± 7.6	0.547^a^
DBP (mmHg)	-1.2 ± 6.7	-0.6 ± 7.3	0.830^a^
HR (beats/min)	-1.0 (-8.5, 16.0)	3.0 (-3.3, 9.0)	0.827^b^
FBG (mmol/L)	0.1 (-0.2, 0.6)	-0.3 (-1.2, 0.2)	0.062^b^
TC (mmol/L)	-0.05 (-0.78, 0.41)	0.03 (-0.21, 0.27)	0.356^b^
TG (mmol/L)	-0.1 (-0.5, 0.2)	-0.1 (-0.4, 0.2)	0.903^b^
LDL (mmol/L)	-0.21 (-0.73, 0.39)	0.00 (-0.31, 0.30)	0.152^b^
HDL (mmol/L)	0.09 ± 0.20	0.04 ± 0.13	0.407^a^
β-HB (mmol/L)	0.01 (-0.03, 0.04)	0.01 (-0.01, 0.03)	0.961^b^
ALT (U/L)	2.0 (-4.5, 8.0)	-2.5 (-5.8, -1.0)	0.075^b^
AST (U/L)	1.0 (-2.5, 6.5)	-2.0 (-6.0, 2.5)	0.233^b^
WBC (10^9/L)	-0.1 ± 0.8	-0.1 ± 0.9	0.957^a^
NEUT%	1.0 ± 6.5	-1.5 ± 6.8	0.339^a^
LYM%	-1.0 ± 5.5	0.2 ± 4.8	0.524^a^
RBC (10^12/L)	0.1 ± 0.4	0.0 ± 0.3	0.802^a^
HB (g/L)	2.7 ± 8.9	-0.6 ± 7.7	0.315^a^
PLT (10^9/L)	3.2 ± 22.1	11.3 ± 25.3	0.388^a^
BFM (kg)	-1.8 ± 2.4	-0.4 ± 1.8	0.119^a^
VFA (cm^2^)	-8.4 ± 12.1	-4.8 ± 16.5	0.523^a^
FMI	-0.7 ± 0.9	-0.2 ± 0.8	0.146^a^
PBF%	-1.0 ± 2.0	-0.5 ± 2.5	0.592^a^
FFM (kg)	-1.1 ± 1.4	0.0 ± 1.6	0.066^a^
SLM (kg)	-1.0 ± 1.2	0.0 ± 1.5	0.055^a^
SMM (kg)	-0.7 ± 0.8	0.1 ± 1.0	**0.042^a^**
TBW (L)	-0.8 ± 1.0	0.0 ± 1.1	0.054^a^
ICW (L)	-0.5 ± 0.6	0.1 ± 0.8	**0.050^a^**
ECW (L)	-0.3 ± 0.4	-0.1 ± 0.4	0.142^a^
AC (cm)	-1.6 ± 1.2	-0.6 ± 1.4	0.068^a^
TSF (cm)	-0.1 ± 0.7	0.0 ± 0.7	0.781^a^
WC (cm)	-1.9 ± 3.2	0.1 ± 3.1	0.110^a^
WHR	0.00 ± 0.03	-0.00 ± 0.04	0.871^a^
Left HGS (kg)	-1.1 ± 3.5	-1.1 ± 2.0	0.936^a^
Right HGS (kg)	-2.0 ± 4.1	-1.2 ± 3.3	0.569^a^
CI	-0.00 ± 0.03	0.01 ± 0.04	0.622^a^

Data are shown as mean ± standard deviation (SD) or median and 25th-75th interquartiles. TRF, time-restricted feeding; CD, control diet; eGFR, estimated glomerular filtration rate; SCR, serum creatinine; BUN, blood urea nitrogen; UA, uric acid; PCR, urinary protein/creatinine; Cys-C, cystatin-C. BMI, body mass index; ALB, albumin; TP, total protein; IL-6, interleukin 6; TNF-α, tumor necrosis factor alpha; CRP, C-reactive protein; Na, serum sodium; P, serum phosphorus; Ca, serum calcium; K, serum potassium; Cl, serum chlorine; SBP, systolic blood pressure; DBP, diastolic blood pressure; HR, heart rate; FBG, fasting blood glucose; TC, total cholesterol; TG, triglycerides; LDL, low-density lipoprotein; HDL, high-density lipoprotein; β-HB, beta hydroxybutyric acid; ALT, alanine transaminase; AST, aspartate transaminase; WBC, white blood cells; NEUT%, neutrophilic granulocyte percentage; LYM%, lymphocyte percentage; RBC, red blood cells; HB, hemoglobin; PLT, platelets; BFM, body fat mass; VFA, visceral fat area; FMI, fat mass index; PBF, percentage of body fat; FFM, fat free mass; SLM, soft lean mass; SMM, skeletal muscle mass; TBW, total body water; ICW, intracellular water; ECW, extracellular water; AC, arm circumference; TSF, triceps skinfold thickness; WC, waist circumference; WHR, waist-to-hip ratio; HGS, handgrip strength; CI, conicity index. Δ represents difference, *p* values < 0.05 are in boldface. ^a^t-test. ^b^Mann-Whitney U test.

### Gut microbiota

One patient in the TRF group did not submit fecal sample at baseline. There was no significant difference in the chao1 index for gut microbiota alpha diversity between the TRF group (Δ = 16.51 ± 64.94) and the CD group (Δ = -6.84 ± 41.62) (*p* = 0.273). When comparing of beta diversity using principal coordinate analysis (PCoA) of the weighted or unweighted UniFra, there was no significant difference between the two groups, either at baseline or after intervention (*p* > 0.05).

**Figure 3:**
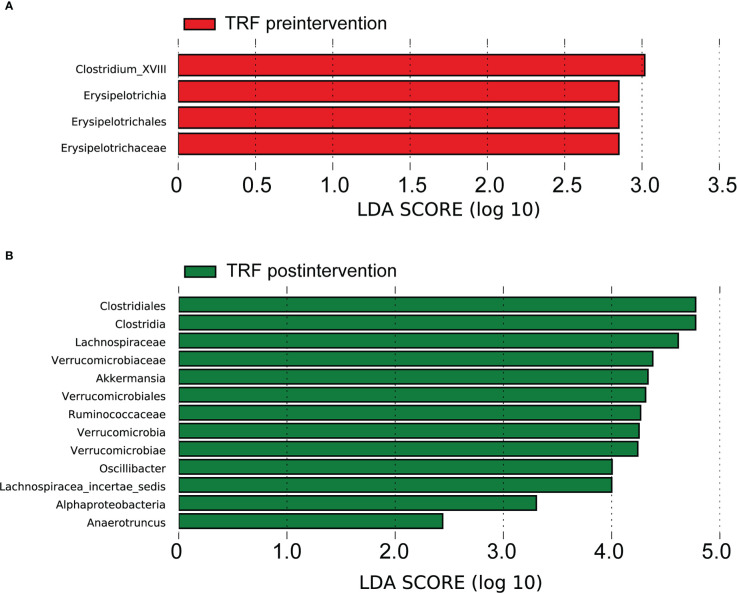
Gut microbiota analysis between groups. TRF, time-restricted feeding; Linear discriminant analysis (LDA) effect size (LEfSe) bar plot; **(A)** difference between groups before the intervention. **(B)** difference between groups after the intervention.

Based on LDA selection, at baseline, from phylum to genus level, there were 4 differential intestinal flora (LAD >2, *p* < 0.05) in the feces of the two groups. Compared with the CD group, the levels of *Erysipelotrichaceae*, *Erysipelotrichales*, *Erysipelotrichia* and *Clostridium_XVIII* in the TRF group had significantly increased (**Figure 3A**).

After the intervention, there were 13 different intestinal flora (LAD >2, *p* < 0.05) in the fecal samples, from phylum to genus level of the two groups, based on LDA analysis. Compared with the CD group, the TRF groups *Lachnospiraceae*, *Lachnospiracea_incertae_sedis*, *Clostridia*, *Clostridiales*, *Verrucomicrobia*, *Verrucomicrobiae*, *Verrucomicrobiales*, *Verrucomicrobiaceae*, *Akkermansia*, *Ruminococcaceae*, *Alphaproteobacteria*, *Oscillibacter* and *Anaerotruncus* significantly increased (**Figure 3B** and **Supplementary Table 2**).

### Adherence; caloric and protein intake

**Figure 4:**
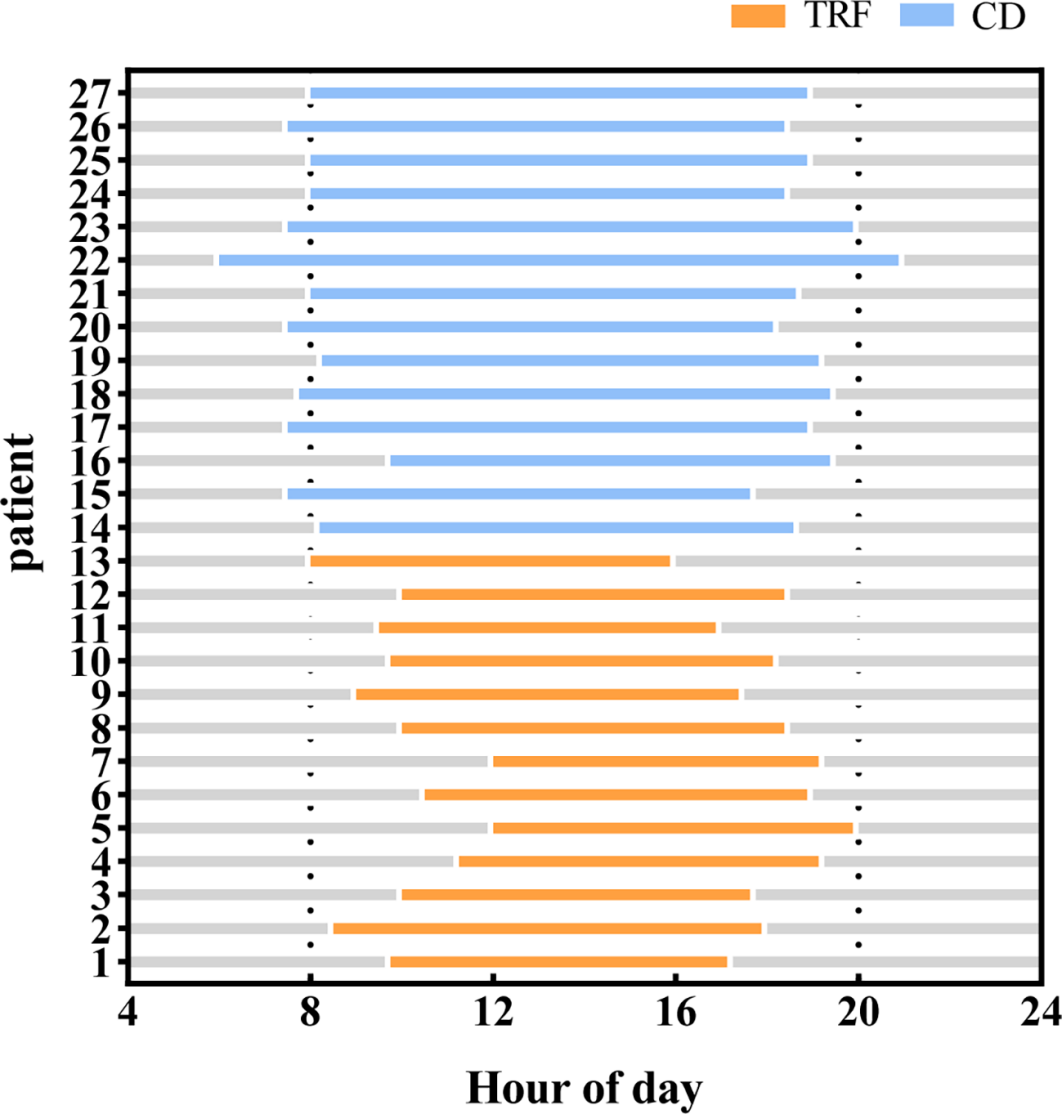
Feeding time between groups. Compared with the CD group, patients in the TRF group had significantly shorter feeding windows.

The TRF group was required to eat three meals within the agreed 8-hour time window, and then fast for 16 hours; there was no feeding time window requirement for the CD group. The feeding time for the TRF group was 8.4 ± 0.7 hours, while that of the CD group was 11.2 ± 1.3 hours (**Figure 4**). Self-reported adherence to the 8-hour feeding window was 84.6% in the TRF group, as measured by diaries, and the compliance was good.

One patient in the CD group did not provide a dietary diary. Caloric intake was declined slightly in the TRF group (21.08 ± 4.36 kcal/kg/d) compared to the CD group (28.39 ± 12.96 kcal/kg/d), although the difference was not statistically significant (*p* = 0.074). Dietary protein intake (DPI) was significantly lower in the TRF group (0.76 ± 0.21 g/kg/d) than in the CD group (1.20 ± 0.53 g/kg/d) (*p* = 0.004). Generally speaking, the DPI of the TRF group generally reflected a low-protein diet, while the CD group did not reach the standard, indicating unsatisfied protein intake.

The intake percentages for the three major macronutrients were similar in both groups. The proportions of protein intake in the TRF group and the CD group were 14.7 ± 3.8% and 17.7 ± 4.1% (*p* = 0.062), respectively. The percentages of high-quality low-protein were 55.70 ± 17.60% and 54.26 ± 11.72% (*p* = 0.397), respectively, while fat and carbohydrates were 26.3 ± 4.1% and 24.6 ± 6.0% (*p* = 0.515), respectively and 60.0 ± 9.8% and 60.0 ± 7.6% (*p* = 0.897), respectively. Overall, dietary intake of water, protein, vitamin B1, vitamin B2, vitamin C, phosphorus, magnesium, zinc, and manganese was significantly lower in the TRF group than in the CD group. Daily macronutrient intakes for both groups are shown in **Table 4**.

**Table 4:** Daily macronutrient intakes, by group, based on dietary records.

Macronutrient	TRF (n = 13)	CD (n = 13)	*p*-value
Energy (kcal)	1178.7 ± 236.4	1496.9 ± 673.4	0.129
Water (g)	561.9 ± 159.3	859.3 ± 416.7	**0.029**
Protein (g)	39.2 (34.6, 50.1)	56.2 (46.7, 74.9)	**0.013**
High-quality low-Protein (g)	23.9 (19.3, 30.4)	36.2 (23.1, 42.1)	0.096
Fat (g)	33.4 ± 6.6	40.6 ± 20.0	0.235
Cholesterol (mg)	269.7 ± 105.4	254.7 ± 111.7	0.727
Carbohydrate (g)	180.1 ± 55.9	224.9 ± 103.5	0.182
Vitamin A (μg)	526.6 ± 207.7	807.5 ± 564.0	0.112
Vitamin B1 (mg)	0.42 ± 0.10	0.83 ± 0.45	**0.006**
Vitamin B2 (mg)	0.54 ± 0.10	0.78 ± 0.32	**0.022**
Vitamin C (mg)	72.3 ± 28.4	149.4 ± 89.8	**0.010**
Vitamin E (mg))	8.2 ± 3.6	10.6 ± 8.2	0.338
Calcium (mg)	292.1 ± 51.9	363.3 ± 177.2	0.186
Phosphorus (mg)	548.8 ± 101.0	869.3 ± 390.0	**0.013**
Potassium (mg)	1156.9 ± 280.0	1699.3 ± 871.1	0.051
Sodium (mg)	1226.7 (933.5, 1,733.7)	1265.0 (626.9, 2,811.7)	0.778
Magnesium (mg)	148.2 ± 32.4	215.2 ± 85.4	**0.018**
Iron (mg)	12.5 (10.2, 15.5)	16.0 (12.9, 24.9)	0.054
Zinc (mg)	5.5 ± 1.5	9.6 ± 4.2	**0.003**
Selenium (μg)	28.6 (22.5, 31.5)	31.5 (25.8, 42.3)	0.158
Copper (mg)	1.0 ± 0.4	1.4 ± 0.6	0.124
Magnesium (mg)	2.2 ± 0.7	3.9 ± 1.6	**0.002**

Data are shown as mean ± standard deviation (SD) or medians (25th-75th interquartiles). TRF, time-restricted feeding; CD, control diet; *p* values < 0.05 are in boldface.

We used multiple linear regression models to evaluate the effect of TRF (**Table 5**). The statistically significance of the grouping was assessed using simple linear regression with delta eGFR as the dependent variable (*p* = 0.049). In the subsequent analysis, the uneven baseline independent variables were included in the multiple linear regression mode. This corrected for baseline confounding factors (*p* = 0.132). To verify the confounding effect of DPI, it was included in the model 3, and the results still suggested grouping remained an independent factor (*p* = 0.048). Then, in model 4, results showed that the regression model was statistically significant (F = 3.508, *p* = 0.018), indicating that after eliminating other factors by multifactorial analysis, the grouping (TRF intervention) had an independent effect on delta eGFR.

**Table 5:** Multiple linear regression analyses.

Model	Unstandardized Coefficients B	Std. Error	Standardized Coefficients Beta	*t*	*p*
Model 1	3.901	1.883	0.383	2.070	**0.049**
Model 2	3.892	2.490	0.382	1.563	0.132
Model 3	5.537	2.646	0.543	2.092	**0.048**
Model 4	4.905	2.165	0.481	2.265	**0.034**

Model 1: grouping.

Model 2: grouping, diastolic blood pressure and fasting blood glucose.

Model 3: grouping, diastolic blood pressure, fasting blood glucose and dietary protein intake (DPI).

Model 4: grouping, diastolic blood pressure, fasting blood glucose, high-density lipoprotein and albumin.

*p* values < 0.05 are in boldface.

### Exercise volume comparison and adverse events

There was no statistically significant difference in the number of walking steps between the two groups. This suggested that there was no significant difference in the daily exercise volume between the two groups (**Supplementary Table 3**).

No deaths or serious adverse events were reported during the study. One patient with stage 4 diabetic nephropathy experienced a slightly hypoglycemic reaction in the TRF group, which resolved with a small amount of food. Moreover, patients in both groups reported the observed 18 discomfortable symptoms. Symptoms that stood out include hunger, fatigue, and satiety. Visual analogue scale (VAS) scores of the discomfort symptoms were similar in both groups at 6 weeks, but hunger in the TRF group was more pronounced than that in the CD group at 12 weeks (*p* = 0.041) (**Supplementary Table 4**).

## Discussion

The preliminary results of this 12-week study showed that compared with the CD group, the eGFR in the TRF group increased by 3.1 ml/min/1.73m^2^ after the intervention. This suggested that the time-restricted feeding therapy may have improved patients’ renal function, and the results correcting for multiple linear regression still showed some benefit. These results were consistent with a study of very low-calorie ketogenic diet intervention in patients with CKD (39). The study included 38 overweight and obese patients with stage 2 CKD, whose eGFR had increased from 76.32 ml/min/1.73m^2^ to 82.21 ml/min/1.73m^2^ after 14 weeks of intervention. Similar results were reported in another intermittent fasting study (40) in which 75% of the 16 patients in the intervention were diabetic kidney disease patients. They experienced an increase in eGFR of 0.4-38.8ml/min/1.72m^2^ after 4-12 months of fasting, also showing some benefit. Other studies have reported inconsistent results. A pilot 2×2 factorial design trial included 111 patients with CKD 3-4 through a 10-15% calorie restriction, combined with exercise for 4 months (14). It reported no significant effect on renal function. Another study followed 8 patients with a low-calorie 500 kcal diet combined with exercise for 12 weeks (16). These results also showed no significant effect on renal function. Most of these studies have been small sample studies with short observation period. Thus, long-term intervention studies with large sample populations are needed to verify the therapy’s clinical efficacy.

We also focused on patient adherence in our study, and from the results, patients in the TRF group experienced some hunger, but within tolerable range, and stated that TRF adherence was good. Likewise, in another similar 2×6 week crossover study involving 24 patients, it was observed that short-term TRF was safe, feasible and well tolerated in healthy middle-aged and elderly adults (41). In addition, although several studies have shown that early time-restricted feeding (eTRF) can provide more significant benefits in controlling blood glucose (42), lipid metabolism (43), and improving mood (44) in obese patients, ending dinner too early is difficult to follow (45). Therefore, in this study, we adopted this specific tactic, requiring patients to start breakfast no later than noon, so that a dietary circadian rhythm could still be maintained. This may have improved patient compliance in the TRF group in this study. Nevertheless, attempting to change a patient’s lifelong eating habits is challenging. In this study, we maintained frequent contact with the patients and the medical staff made efforts to motivate the patients. This behavioral intervention may have improved the patients’ determination to control their weight, enabling patients to maintain better dietary compliance and benefit from it (46).

In this study, we tried to explain the underlying mechanism of TRF’s benefit for CKD patients. We believe that it may be due to the following reasons.

The first is the change in body composition. Our study reported a significant decrease in body weight and BMI in the TRF group. At the same time, the body composition analysis indicated not only a reduction in adiposity (body fat mass and visceral fat), but also a parallel reduction in muscle and water. It showed that patients in the TRF group were more likely to reduce protein intake and calorie intake when they followed TRF and a low-protein diet. This reduced the burden on the kidneys and improved renal outcomes. The renal risks of obesity include renal function impairment and damage to renal structure (47). Many studies have shown that higher BMI increases the risk of hypertension and diabetes in CKD patients, and increases the incidence of end-point events. Meanwhile, weight loss through lifestyle changes or bariatric surgery may improve renal outcomes (48). Therefore, our study provides another feasible weight loss approach that may lead to favorable outcomes in overweight and obese patients with CKD. These results also suggest that TRF may lead to too much reduction in CKD patient intakes. Reduced protein intake may lead to sarcopenia, decreasing muscle strength, which in turn could lead to disease progression and increased mortality (49). Therefore, TRF should be accompanied by as much exercise as possible for patients to maintain muscle mass. Although our patients did not engage in weekly high-intensity exercise or resistance training according to the guidelines (50), all patients reported sustaining walks at least 3-5 times per week consisting of 7,415 (3,887, 12,597) steps per day. This may have been why although the body composition of the patients in the TRF group changed, there was no significant change in muscle strength (handgrip strength).

Secondly, TRF may cause changes in the gut microbiota. Although our study did not affect the species diversity of gut microbiota, TRF induced positive changes in the gut microbiota. Significant elevation of harmful gut microbiota was observed in the TRF group before the intervention. Yet in the limited studies, it was not enough to summarize the role and influence of *Clostridium_XVIII*, which may be related to cholesterol metabolism. *Clostridium_XVIII* may be positively correlated with body weight and BMI (51, 52). The enrichment of *Erysipelotrichaceae* is associated with metabolic disorders and metabolic syndrome. In a study of obese women, the abundance of *Erysipelotrichaceae* was found to be higher in the obese group and in the group with obesity combined with metabolic syndrome compared to healthy controls (53). Results of another study analyzing the gut microbiota of immunoglobulin A nephropathy (IgAN) showed that the risk gene *rs6065904* in patients was positively correlated with the abundance of *Erysipelotrichaceae*, which is thought to impair intestinal health leading to disease (54). After TRF intervention, beneficial gut microbiota that helped to improve metabolism increased significantly. As is known, *Akkermansia*(*AKK*), *Oscillibacter*, and *Anaerotruncus* are in the phylum *Verrucomicrobia*. *AKK* can prevent and treat obesity (55), maintain energy and metabolic homeostasis (56), and repair the intestinal barrier (57). Consistent with our previous observations, fasting methods such as intermittent fasting can significantly increase *AKK* abundance (58–60). *AKK* is negatively correlated with fasting blood glucose, waist-to-hip ratio, and subcutaneous adipocyte diameter. Subjects with higher *AKK* abundance showed a healthier metabolic status and similarly showed substantial improvements in insulin sensitivity, as well as other clinical indicators after caloric restriction intervention (61). *AKK* supplementation resulted in a decrease in total cholesterol and a trend toward a reduction in body weight, body fat mass, and hip circumference (62). Like *AKK*, *Oscillibacter* is listed as a next-generation probiotic candidate because of its ability to produce short-chain fatty acids such as butyrate. Several studies have shown that *Oscillibacter* is highly correlated with obesity and metabolic disorders. A significantly lower abundance of *Oscillibacter* among obese patients is positively correlated with leanness and health (63), and correlated with a lower risk of metabolic syndrome, lower triglycerides, lower fasting glucose, and lower homeostatic model assessment of insulin resistance (64). The genus *Anaerotruncus* is more abundant in lean people and negatively correlated with BMI (65). Thus, we inferred that the changes in gut microbiota were due to TRF’s positive influence, which may be more conducive to the improvement of various metabolic factors in CKD patients.

The third reason may be inflammatory factors. However, we observed no conclusive positive results in our study. We observed the effect of TRF on the inflammatory factors TNF-α, IL-6 and CRP, but they showed different tendencies separately. Although animal studies have shown that TRF can reduce the inflammatory factors TNF-α and IL-6 (66), only a few intermittent fasting measures in human trials have decreased TNF-α, IL-6, IL-β and IL-10 (15, 67), while more of the literature has reported no effect on inflammatory factors (68–71). This is consistent with the results of our study. The reason for this may be a long-term vicious cycle between excessive fat accumulation, chronic inflammation and insulin resistance. We speculate that the TRF intervention may require more time to be more beneficial to reduce inflammation and insulin resistance.

In addition, in our study, we also observed a decrease in serum phosphate and uric acid, maintenance of total protein and albumin, in the TRF group. This may have been attributed to a significant decrease in patients’ dietary intake, with reduced phosphorus, protein and purine intake which may also lead to further approval of renal outcomes.

To our knowledge, this is the first study to evaluate the effects of TRF in overweight and obese moderate-to-severe CKD in China, with low drop-out rates, and specifically assessing dietary intake and gut microbiota. Nonetheless, our exploratory study has several limitations. Firstly, due to the specificity of the intervention and the impact of COVID-19, this study is merely a small sample size study. Although only 28 patients were ultimately included in the study, the patients were representative. They were sourced from outpatient clinics and recruited and included in strict accordance with the study criteria without special selection. However, because of the relatively small sample size, the conclusions we drew were cautious. Due to the small sample and imperfect compliance, it may not reflect the true effect of TRF intervention. Additionally, this was a non-randomized controlled study, and there may be some variation in individuals’ self-discipline regarding their lifestyles and such as dietary control between the two groups. This may have introduced bias into the analysis of the two groups’ results, but may be helpful in terms of adherence. Finally, because of the short duration of the intervention in this study, and the lack of follow-up for patients in both groups, we could not determine the long-term efficacy of TRF therapy.

## Conclusions

Our study suggests that TRF may help improve renal function in overweight and obese moderate-to-severe CKD patients. This may be due to the patients’ weight loss, stable nutritional status, and the increase in beneficial bacteria in the gut microbiota. It may also have been due to the decrease in uric acid and serum phosphorus levels. Patients maintained good compliance and clinical implementation reliability under strict monitoring by health care professionals. The results suggest that TRF may be a safe and effective dietary intervention for overweight and obese CKD patients. However, long-term, large-scale randomized clinical trials are needed for further validation.

## Data availability statement

The raw sequence data reported in this paper have been deposited in the Genome Sequence Archive (Genomics, Proteomics & Bioinformatics 2021) in National Genomics Data Center (Nucleic Acids Res 2022), China National Center for Bioinformation/Beijing Institute of Genomics, Chinese Academy of Sciences (accession number GSA-Human: HRA004144), publicly accessible at https://ngdc.cncb.ac.cn/gsa-human.

## Ethics statement

The studies involving human participants were reviewed and approved by Ethics Committee at the Guangdong Provincial Hospital of Traditional Chinese Medicine. The patients/participants provided their written informed consent to participate in this study. Written informed consent was obtained from the individual(s) for the publication of any potentially identifiable images or data included in this article.

## Author contributions

Conceptualization: B-NL and Y-FW. Methodology: B-NL, Y-FW and X-SL. Formal analysis: B-NL and W-WO. Investigation: B-NL, J-HL, X-YX, L-ZF, FT, X-ZX, M-TW, B-JX and L-YC. Resources: L-ZF, FT, Y-FW and X-SL. Data curation: B-NL, J-HL, X-YX. Writing—original draft preparation: B-NL. Writing—review and editing: J-HL, X-YX, W-WO and Y-FW. Supervision: Y-FW and X-SL. Funding acquisition: Y-FW and X-SL. All authors have read and agreed to the published version of the manuscript. All authors contributed to the article and approved the submitted version.

## Footnotes

1
http://www.chinacdc.cn/jkzt/yyhspws/xzdc/201708/P020170807451756522862.pdf
