# Supplementary material for: Time-restricted feeding’s effect on overweight and obese patients with chronic kidney disease stages 3-4: A prospective non-randomized control pilot study
Source: Front Endocrinol (Lausanne). 2023 Mar 22;14:1096093. doi: 10.3389/fendo.2023.1096093 (PMC10111616; doi:10.3389/fendo.2023.1096093)
Supplement: Supplementary file 1 [file DataSheet_1.pdf]

Supplementary Material

Supplementary Figure 1.

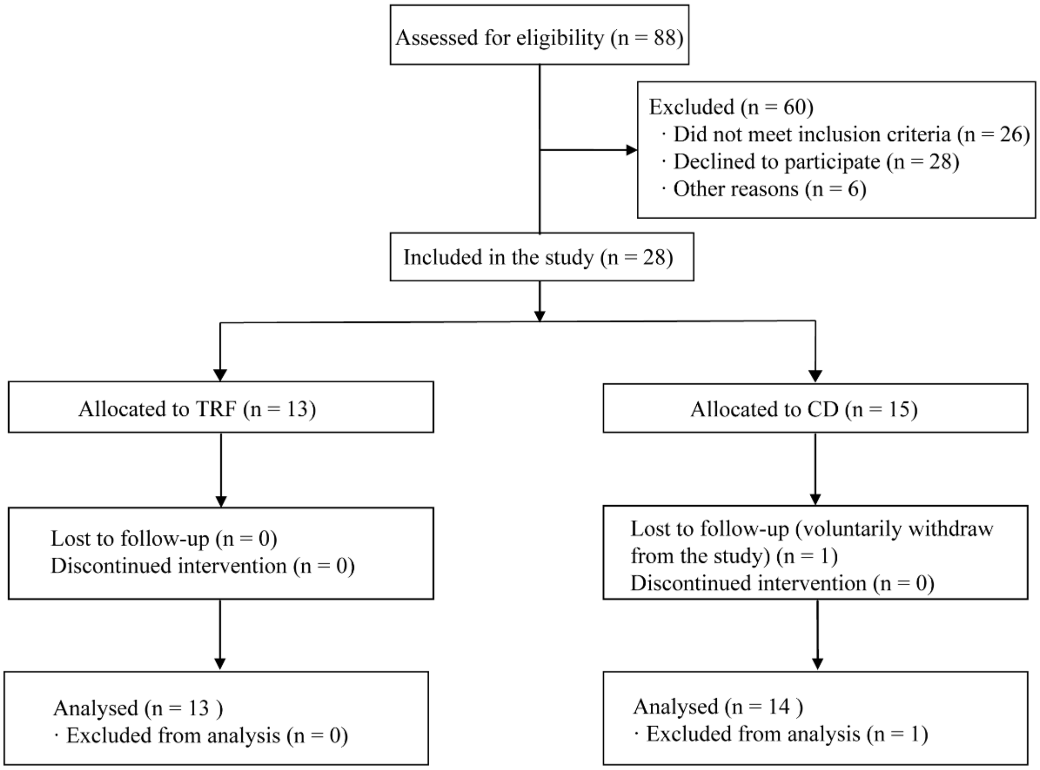

Supplementary Figure 1. Study flow diagram.

Supplementary Table 1. Important biochemical indicators and reference value

Variable	Reference value
Serum creatinine (μmol/L)	41 - 81
Blood urea nitrogen (mmol/L)	3.10 - 8.80
Uric acid (μmol/L)	155 - 357 (female), 208 - 428 (male)
Cystatin-C (mg/L)	0.54 - 1.15
Albumin (g/L)	40.0 - 55.0
Total protein (g/L)	65.0 - 85.0
Alanine transaminase (U/L)	7 - 40
Aspartate transaminase (U/L)	13 - 35
Total cholesterol (mmol/L)	3.38 - 5.2
Triglycerides (mmol/L)	0.55 - 1.7
Low-density lipoprotein (mmol/L)	0.00 - 3.37
High-density lipoprotein (mmol/L)	> 1.15

Interleukin 6 (pg/mL)	0.00 - 7.00
Tumor necrosis factor alpha (pg/ml)	≤ 8.10
C-reactive protein (mg/L)	0.00 - 6.00

Supplementary Table 2. Different intestinal flora between the two groups, before and after treatment

	Microbiota	LAD	*p*-value
Before intervention	*Erysipelotrichaceae*	2.85	**0.031**
	Clostridium_XVIII	3.02	**0.003**
	Erysipelotrichales	2.85	**0.031**
	Erysipelotrichia	2.85	**0.031**
After intervention	*Lachnospiracea_incertae_sedis*	4.03	**0.047**
	Akkermansia	4.56	**0.028**
	Ruminococcaceae	4.27	**0.029**
	Alphaproteobacteria	3.41	**0.028**
	Oscillibacter	3.98	**0.027**
	Verrucomicrobiae	4.52	**0.028**
	Clostridia	4.75	**0.015**
	Lachnospiraceae	4.57	**0.013**
	Verrucomicrobiales	4.48	**0.028**
	Clostridiales	4.75	**0.015**
	Verrucomicrobiaceae	4.49	**0.028**
	Anaerotruncus	2.21	**0.016**
	Verrucomicrobia	4.46	**0.028**

p values < 0.05 are in boldface.

Supplementary Table 3. Walking steps for patients in both groups

Step count	TRF (n = 13)	CD (n = 14)	*p*-value
Weekday step count 1	8339(3,988, 9,782)	5399(4,059, 10,000)	0.770
Weekday step count 2	7500(3,173, 10,0000)	4250(2,875, 10,000)	0.559
Weekend step count	8565(5,696, 10,500)	6741(4,009, 11,426)	0.733

Data are shown as medians (25th-75th interquartiles). TRF: time-restricted feeding; CD: control diet.

Supplementary Table 4. Discomfort symptoms VAS scores for both groups

Symptoms	6 weeks			12 weeks		
	TRF (n = 13)	CD (n = 14)	*p*-value	TRF (n = 13)	CD (n = 14)	*p*-value
hunger	4.0 (0.5, 5.0)	1.0 (0.8, 4.0)	0.167	3.0 (1.5, 6.0)	1.0 (0.0, 3.3)	**0.041**
fatigue	2.0 (0.0, 5.0)	1.0 (0.0, 3.3)	0.458	2.0 (0.0, 4.0)	2.0 (0.0, 4.3)	0.980
satiety	4.0 (2.5, 4.5)	1.5 (0.8, 3.0)	0.055	2.0 (0.0, 2.5)	1.0 (0.0, 2.3)	0.312

flatulence	0.0 (0.0, 3.0)	1.5 (0.0, 3.3)	0.202	0.0 (0.0, 2.5)	0.5 (0.0, 2.0) *	0.662
nausea	0.0 (0.0, 1.5)	0.5 (0.0, 1.0)	0.311	0.0 (0.0, 0.0)	0.0 (0.0, 0.0)	0.387
vomiting	0.0 (0.0, 0.0)	0.0 (0.0, 1.0)	0.222	0.0 (0.0, 0.0)	0.0 (0.0, 0.0)	0.657
constipation	0.0 (0.0, 5.0)	0.5 (0.0, 2.5)	0.753	0.0 (0.0, 0.0)	0.0 (0.0, 2.3)	0.506
diarrhea	0.0 (0.0, 0.5)	0.0 (0.0, 0.3)	0.268	0.0 (0.0, 0.0)	0.0 (0.0, 1.0)	0.268
bad breath	0.0 (0.0, 1.5)	1.0 (0.8, 3.3)	0.133	0.0 (0.0, 1.0)	1.0 (0.0, 2.0)	0.133
dry mouth	2.0 (0.0, 5.0)	1.0 (0.0, 2.3)	0.278	0.0 (0.0, 2.5)	0.5 (0.0, 1.0) *	0.957
dizziness	0.0 (0.0, 4.0)	0.0 (0.0, 1.0)	0.750	0.0 (0.0, 0.5)	0.0 (0.0, 1.0)	0.507
headache	0.0 (0.0, 1.5)	0.0 (0.0, 1.0)	0.809	0.0 (0.0, 0.0)	0.0 (0.0, 1.0)	0.809
weakness	1.0 (0.0, 5.5)	0.0 (0.0, 1.0)	0.066	0.0 (0.0, 1.5)	0.0 (0.0, 1.3)	0.954
irritability	0.0 (0.0, 3.0)	0.0 (0.0, 1.0)	0.502	0.0 (0.0, 1.5)	0.0 (0.0, 2.0)	0.338
unhappiness	0.0 (0.0, 3.5)	0.0 (0.0, 1.0)	0.377	0.0 (0.0, 1.5)	0.0 (0.0, 1.0)	0.593
depression	0.0 (0.0, 1.5)	0.0 (0.0, 0.3)	0.815	0.0 (0.0, 0.5)	0.0 (0.0, 1.0)	0.900
anxiety	0.0 (0.0, 1.5)	0.0 (0.0, 1.0)	0.975	0.0 (0.0, 1.5)	0.0 (0.0, 1.0)	0.718
overeating	0.0 (0.0, 1.5)	0.0 (0.0, 1.5)	0.505	0.0 (0.0, 2.0)	0.0 (0.0, 1.0)	0.935

Data are shown as medians (25th-75th interquartiles). VAS: visual analogue scale; TRF: time-restricted feeding; CD: control diet. * represents a significant difference between the CD group before and after 6 and 12 weeks. p values < 0.05 are in boldface.
